# Barriers to accessing neurosurgical care in low- and middle-income countries from Africa: editorial

**DOI:** 10.1097/MS9.0000000000001758

**Published:** 2024-01-25

**Authors:** Inibehe I. Okon, Aymar Akilimali, Muhammad Furqan, Fadele K. Precious, Tolulope J. Gbayisomore, Oday Atallah, Micheal O. Erhayanmen, Ekpenyong C. Christopher, Florence Umutoni, Menelas Nkeshimana, Don E. Lucero-Prisno

**Affiliations:** a Department of Research, Medical Research Circle (MedReC), Bukavu, DR Congo; b College of Medicine, University of Uyo, Akwa Ibom; c College of Medicine, Ambrose Alli University, Ekpoma; d College of Medicine, University of Nigeria, Nsukka, Enugu State; e Data Intelligence and Innovation Unit, University of Benin Teaching Hospital, Benin City, Edo State, Nigeria; f Department of Neurosurgery, Hannover Medical School, Hannover, Germany; g Faculty of Medicine, King Edward Medical University, Lahore, Pakistan; h Faculty of Medicine, University of Rwanda; i Department of Health Workforce Development, Ministry of Health, Kigali, Rwanda; j Department of Global Health and Development, London School of Hygiene and Tropical Medicine, London, United Kingdom

## Introduction

Among various fundamental human rights, ensuring equal access to healthcare services for all individuals is a core fundamental human right. On Human Rights Day 2017, in accordance with the Constitution of the WHO, it was asserted that the fundamental right to health implies that every individual should have the opportunity to obtain necessary health services without facing financial adversity, ensuring accessibility based on need, time, and location^1^. However, delivering advanced healthcare, particularly in fields like neurosurgery, encounters obstacles. A recent report in The Lancet highlighted that ~5 billion people worldwide lack basic surgical care, with a significant impact on low-income and middle-income countries (LMICs)^2^. Neurosurgical diseases impose a substantial burden on LMICs, with middle-income countries bearing two-thirds of the global burden of hemorrhagic strokes^3^. The contrast in resources between LMICs and high-income countries results in an unmet demand for ~5 million neurosurgical cases each year^4^. The aim of this study was to characterize the practice of neurosurgery in Africa and identify the obstacles to accessing neurosurgical care in LMICs within the African region.

## The systemic challenges and barriers

Previous research has pinpointed several factors contributing to the existing difficulties in ensuring adequate surgical care in Africa. These include lack of financial resources and the required equipment, an inadequate healthcare infrastructure, and a scarcity of neurosurgeons. Presently, most LMICs are having difficulties in this regard^5^. Approximately 25.3% of the population in sub-Saharan Africa has access to neurosurgery services within 2 h, with a workforce density of 1:4 000 000 in Africa^6,7^. The lack of standardized guidelines for neurosurgical techniques and management protocols, surgical technique ignorance, limited exposure to intra-operative real-time decision-making, and a scarcity of guidelines for optimal postoperative care are the main causes of decision-making difficulties^8^. These issues further complicate the delivery of neurosurgical services to already underserved populations. Despite the noticeable difference, access to neurosurgical services is paradoxically small in many regions of Africa because of the apparent awful discrepancy in earnings. Eighty-five percent of Africans live on <6 dollars a day, and because the expenses for surgical care are generally out of pocket, the limitation of neurosurgical care potentially partakes to financial impoverishment^9^. The price of neurosurgical care team continues to be a critical obstacle, albeit reasonably economical but significantly amounting to as high as 40.18% of GDP per capital^10^. However, in some cases, patients do not seek neurological services because of the actual awareness that these services are too costly to afford^11^. This myth may appear insignificant but no less contributes to barriers to accessing neurosurgical services.

Meanwhile, an important factor in determining obstacles to obtaining neurosurgical care is healthcare-seeking behavior (HSB) which is defined as ‘any action or inaction undertaken by individuals who perceive themselves to have a health problem or to be ill to find an appropriate remedy’^12^. A person’s traits and behaviors, the physical surroundings, the socioeconomic environment, the expense of healthcare, and accessibility all influence HSB^12^. For instance, patients from Ethiopia and Nigeria seek treatment from traditional healers or spiritual healers before seeing neurosurgeons because they consider cranial disorders to be supernatural^12,13^, which frequently results in patients showing up later in the course of the illness, which can make treatment choices more difficult and decrease the efficacy of available interventions. Thus, unwarranted HSBs and misperceptions about neurosurgery are significant obstacles to receiving care.

## Conclusion

In conclusion, the editorial highlights formidable barriers to neurosurgical care in African LMICs, including financial constraints, healthcare infrastructure gaps, and a shortage of neurosurgeons. These challenges create a substantial deficit in providing essential services to underserved populations, aggravated by economic disparities, and misconceptions hindering timely access to care.

## Recommendations

1. Policymakers and healthcare authorities in African countries should collaborate to formulate and implement policies aimed at addressing the systemic challenges identified. This includes investing in healthcare infrastructure, increasing the number of neurosurgeons, and developing standardized guidelines for neurosurgical practices.
2. Financial support mechanisms, such as insurance schemes or government subsidies, should be introduced to alleviate the financial burden on patients seeking neurosurgical care. This can help ensure that individuals, particularly those with lower incomes, can access essential neurosurgical services without facing undue financial hardship.
3. Community engagement and educational campaigns should be launched targeted to dispel myths and misconceptions surrounding neurosurgical care to specific sub-regions and communities. Through awareness and understanding of the importance of timely intervention, healthcare-seeking behaviors can be positively influenced, leading to earlier and more effective access to neurosurgical services.
4. Collaborative research efforts and partnerships between medical schools, healthcare institutions, researchers and international organizations to better understand and address the unique challenges faced by LMICs in Africa regarding neurosurgical care. This can lead to innovative solutions and interventions tailored to the specific needs of the sub-regions in Africa.

## Ethical approval

Ethics approval was not required for this editorial.

## Consent

Informed consent was not required for this editorial.

## Sources of funding

The authors did not receive any financial support for this work. No funding has been received for the conduct of this study.

## Author contribution

All authors contributed equally.

## Conflicts of interest disclosure

The authors declare that there is no conflicts of interest.

## Research registration unique identifying number (UIN)

Not applicable.

## Guarantor

Inibehe Ime Okon and Aymar Akilimali.

## Data availability statement

Not applicable.

## Provenance and peer review

Not commissioned, externally peer-reviewed.
